# Total Saponins from *Nigella glandulifera* Seeds Ameliorate Adjuvant-Induced Rheumatoid Arthritis in Rats by Inhibition of an Inflammatory Response and Bone Erosion

**DOI:** 10.1155/2021/6613527

**Published:** 2021-01-28

**Authors:** Li Zeng, Chenyang Li, Hailun Jiang, Yan Chen, Zhuorong Li, Fang Xu, Rui Liu

**Affiliations:** ^1^Institute of Medicinal Biotechnology, Chinese Academy of Medical Sciences and Peking Union Medical College, Beijing 100050, China; ^2^Key Laboratory of Uighur Medicine of Xinjiang Uygur Autonomous Region, Xinjiang Institute of Materia Medica, Urumqi 830004, China

## Abstract

Rheumatoid arthritis (RA) is a widespread inflammatory disease whose clinical manifestations are joint swelling, pain, and disability, affecting approximately 1% of individuals worldwide. Conventional anti-RA drugs currently used in clinic have severe side effects. The present study is aimed at investigating the antiarthritic effects of total saponins from *Nigella glandulifera* seeds (TSNGS) in rats with adjuvant-induced rheumatoid arthritis (AIA). Arthritis score, paw swelling, and body weight were monitored throughout the period of TSNGS treatment. The histopathological features and levels of cytokines, including IFN-*γ*, TNF-*α*, IL-1*β*, IL-4, IL-6, IL-10, and IL-17A, and OPG/RANKL signaling, were measured to determine the amelioration by TSNGS and its potential mechanisms on the inflammatory response and bone erosion. The differentiation of regulatory T cells (Tregs) in serum was assessed by flow cytometry. The results demonstrate that TSNGS at 10 mg/kg, 50 mg/kg, and 250 mg/kg inhibited AIA-induced clinical score, paw swelling, and histological changes. TSNGS reduced the immune-inflammatory reaction by restoring the secretion and expression of inflammatory cytokines and elevating the proportion of CD4^+^ CD25^+^ Tregs, accompanied by an increase in transcription factor Foxp3 levels. TSNGS also displayed bone protection by upregulation of the OPG/RANKL pathway. Collectively, TSNGS inhibited arthritis in AIA rats and so represents a potential novel treatment for RA.

## 1. Introduction

Rheumatoid arthritis (RA) is a chronic and progressive autoimmune disease of unknown etiology, characterized by synovial inflammation and hyperplasia, the formation of pannus, and cartilage destruction that result in irreversible joint damage and severe disability [1]. It has been reported that the prevalence of RA is between 0.4% and 1.2%, while the incidence in women is almost 2-4-fold greater than in males [2, 3]. The focus of treatment given to RA patients is currently immune suppression plus nonsteroidal anti-inflammatory drugs (NSAIDs) and disease-modifying antirheumatic drugs (DMARDs) to relieve their immuno-inflammatory response and the pain symptoms of RA, including Tripterygium wilfordii polyglycosides, methotrexate, and glucocorticoids. Long-term use leads to serious side effects on the kidney, bone, stomach, and other tissues and organs. As a result, the development of a drug that cures RA is urgently required for clinical use.

Traditional Chinese Medicine (TCM), especially herbal medicine, can be used to a great extent to improve RA treatment by utilizing the synergistic effects of herbal components and neutralizing their toxic effects in the mixture. Of the effective clinical formulae, Gui-Zhi-Shao-Yao-Zhi-Mu decoction, developed by Zhongjing Zhang with extensive use in RA treatments in China from the Han Dynasty, demonstrates therapeutic properties with analgesic and anti-inflammatory effects, in addition to the regulation of an antigen-driven autoimmune response, whole-body lipid metabolism, and matrix degradation in human joints [4]. Another TCM formula, the Bi-Qi capsule, commonly prescribed for RA in China, alleviates RA-induced inflammation, synovial hyperplasia, and cartilage destruction [5]. Many extensively studied natural products used for the therapy RA, such as sinomenine [6], curcumin [7], resveratrol [8], and gingerol [9], are reported to have the capability to suppress numerous proinflammatory mediators. Therefore, herbal products possessing beneficial properties may serve as potential therapeutic agents to treat RA, either alone or in combination with particular mainstream antiarthritic drugs.

The initiation of RA is not yet fully understood, but it has been confirmed that dysregulation of the immuno-inflammatory system plays a significant role in the pathophysiology of RA [10]. The aberrant production of inflammatory mediators such as TNF-*α*, IL-1*β*, IL-6, IL-4, and IL-17 secreted by a variety of immunocytes and macrophages infiltrate into the synovium of multiple joints, leading to long-term inflammation, ultimately resulting in irreversible joint and cartilage destruction [11]. Given the crucial role that proinflammatory cytokines have in the induction and maintenance of RA, regulation of these cytokines on regulatory T cells (Tregs) can be a target to strengthen immune tolerance and protect against the formation of osteoclasts [12]. Synovitis is the principal cause of joint bone erosion, closely associated with lack of control of a number of regulators that maintain osteoclast homeostasis, such as increased receptor activator of nuclear factor 𝜅B ligand (RANKL) levels and decreased osteoprotegerin (OPG) [13, 14]. Thus, the main purpose of current treatments for RA is to reduce the immuno-inflammatory response, inhibit the development of lesions and bone damage, and protect the function of joints.

*Nigella glandulifera* seeds belong to the buttercup family Ranunculaceae. As a traditional Uighur medicine, they are widely used for hypertension, diabetes, and inflammatory diseases such as bronchitis, arthritis, and hepatitis and exhibit considerable efficacy with few side effects [15]. Contributions to the medical properties of *Nigella glandulifera* seeds are the many active constituents, of which saponins are the principal component in extracts of water, comprising 64.5% of the total. Total saponins from *Nigella glandulifera* seeds (TSNGS) have been reported to participate in providing their anti-inflammatory, analgesic, and antioxidant properties [16, 17]. We have previously reported on the potent effects of TSNGS on reducing paw swelling and inflammation in a mouse model of collagen-induced arthritis [18]. However, there still lacks evidence for the pharmacological mechanism for the therapeutic effects of TSNGS against RA. In the present study, we have investigated the therapeutic effects of TSNGS in an adjuvant-induced rheumatoid arthritis (AIA) rat model and explored the potential mechanism by which immuno-inflammatory function is recovered and bone erosion diminished.

## 2. Materials and Methods

### 2.1. Chemical and Reagents

TSNGS was provided by the Xinjiang Institute of Materia Medica (Urumqi, China). Tripterygium glycosides (TG) were purchased from the Hunan Qianjin Xieli Pharmaceutical Co. Ltd. (Zhuzhou, China).

### 2.2. Plant Materials and Preparation of TSNGS

**Figure 1:**
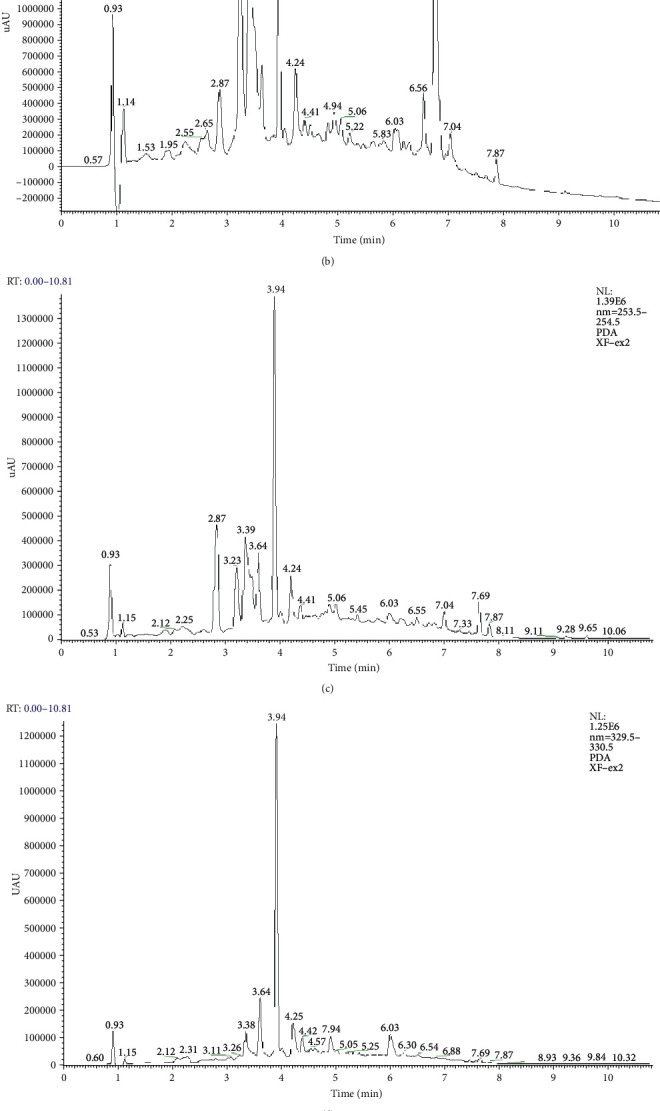
Analysis of the UHPLC-HRMS chromatogram of TSNGS. The chemical composition of TSNGS was confirmed by UHPLC-HRMS based on retention time and fragmentation behavior, in addition to accurate mass measurement and characteristic fragmentation. A total of 21 compounds were identified, including triterpene saponins, alkaloids, flavonoid glycosides, and phenolic compounds. (a) UHPLC-Q-Orbitrap-HRMS chromatogram of TSNGS. UHPLC-PDA chromatogram of TSNGS at the wavelengths: (b) 210 nm, (c) 254 nm, (d) 330 nm, and (e) 365 nm.

*Nigella glandulifera* seeds were collected from Hetian (Xinjiang, China) in 2017. The strain was authenticated by Prof. Jiang He, Xinjiang Institute of Materia Medica. A voucher specimen (No. NG20170830) was deposited in the Herbarium of Xinjiang Institute of Material Medica (Urumqi, China). The extraction of TSNGS has been described in our previously published report [17]. Qualitative analysis was performed using a UHPLC-Q-Exactive mass spectrometry system consisting of an Ultimate 3000 RSLC (Dionex, USA) combined with a Quadrupole-Orbitrap-HRMS (Thermo Fisher Scientific, Bremen, Germany) (Figure 1).

### 2.3. Animals and Treatment

Wistar specific-pathogen-free (SPF) rats (6-7 weeks old, mean body weight 180 ± 10 g, thirty males and thirty females) were provided by the Beijing Vital River Laboratory Animal Technology Co. Ltd (Beijing, China). The rats were maintained in standard laboratory conditions (21°C-25°C, 40%-60% humidity within a 12 h light/12 h dark cycle) with free access to standard rat chow and tap water, in accordance with the Experimental Animal Care and Use Committee guidelines. The Animal Care and Use Ethics Committee of the Institute of Medicinal Biotechnology, Chinese Academy of Medical Sciences and Peking Union Medical College approved all animal experiments (No. IMB-201908-D6).

The AIA model is a well-established chronic inflammatory model, widely used for the study of diverse human arthritis, especially rheumatoid arthritis. The AIA rat model was established in accordance with previously published literature [19]. Briefly, 50 AIA rats were injected once with 0.1 mL complete Freund's adjuvant (CFA) (1 mg/mL; Sigma-Aldrich, Merck KGaA, Darmstadt, Germany) intradermally into the hind paw on day 1 of the experiment. Ten control rats were injected with the same volume of normal saline. The rats were randomly divided into a control group, AIA model group, AIA treatment groups, in which rats were administered 10 mg/kg, 50 mg/kg, or 250 mg/kg TSNGS intragastrically, and AIA positive control group, which received the drug TG (9 mg/kg). There were ten rats in each group (five males and five females). All treated AIA rats were administrated TSNGS or TG dissolved in 0.5% sodium carboxymethyl cellulose (CMC-Na) by oral gavage once per day from the 3rd to the 21st day. The control group and AIA model group received 0.5% CMC-Na by gavage over the same period.

The rats were sacrificed by inducing deep anesthesia using intraperitoneal injection of 60 mg/kg pentobarbital. Blood was obtained by cardiac puncture. Serum was obtained from the blood by centrifugation at 1000 g for 15 min after which it was stored at -80°C for the assessment of inflammatory cytokines. A proportion of the blood was anticoagulated in an ethylenediamine tetraacetic acid- (EDTA-) coated anticoagulation tube at 4°C for the evaluation of Tregs. Ankle joints and knee joints were harvested from 6 rats in each group, three males and three females, for histopathological analysis. The ankle joints, knee joints, and synovial tissues of the remaining rats were collected for Western blot (WB) analysis and quantitative polymerase chain reaction (qPCR).

### 2.4. Evaluation of Paw Edema, Arthritis Score, and Body Weight in AIA Rats

Paw edema, arthritis score, and body weight were recorded to evaluate the severity of the RA pathological process. Prior to CFA injection, the volume of the hind paw was measured as the baseline value. The first CFA injection was administered on day 1. The same measurements were performed on days 3, 5, 7, 9, 11, 13, 15, 17, 19, and 21 following CFA injection. The clinical paw score of the AIA model was recorded independently by two observers blinded to the grouping of each rat in accordance with previous research (0 = normal, 1 = slight swelling and redness, 2 = moderate swelling and redness, 3 = severe swelling and redness to the ankle, and 4 = extremely severe swelling and redness representing severe deformity) [20]. In addition, the weight of each rat was recorded (in grams (g)) every two days from the 3rd day.

### 2.5. Histochemical and Immunohistochemical Evaluation

The knee and ankle joints were harvested from rats on the 21st day after injection of CFA. The specimens were fixed in 4% paraformaldehyde for 48 hours, decalcified in buffered 10% EDTA at 4°C for 1 month, then embedded in paraffin. Tissues were serially sectioned onto standard glass slides at a thickness of 4 *μ*m then subsequently deparaffinized and stained with hematoxylin and eosin (H&E, Servicebio, Wuhan, China). Image acquisition, morphological changes, and cellular infiltration analysis were conducted after dehydration and mounting. Notably, semiquantitative grading with five scores was used to evaluate the histopathological changes in the AIA joints (0 = insignificant changes, 1 = minimal change, 2 = mild changes, 3 = moderate changes, and 4 = marked changes).

Tissue sections were created for evaluation by immunohistochemistry and incubated with anti-forkhead box p3 (Foxp3) antibody (Servicebio) at 4°C overnight, a color reaction developed using diaminobenzidine tetrahydrochloride (DAB), and nuclei counterstained with hematoxylin in accordance with the manufacturer's instructions. Images were acquired using an inverted microscope (Thermo Fisher Scientific, Carlsbad, CA, United States) and the ImageJ software used for qualification (National Institutes of Health, Bethesda, MD, United States).

### 2.6. qPCR Analysis

The expression of tumor necrosis factor-alpha (TNF-*α*), interleukin-1*β* (IL-1*β*), interleukin-6 (IL-6), interleukin-10 (IL-10), and interleukin-17A (IL-17A) at the mRNA level was quantified by qPCR, in accordance with standard protocols. Firstly, total RNA was isolated from rat synovial tissue using a Cell/Tissue Total RNA Isolation Mini kit (Vazyme Biotech, Nanjing, China), of which 100 ng was reversed transcribed to complementary DNA (cDNA) using a HiScript II First Strand cDNA Synthesis kit combined with an amplification procedure in the thermocycler as follows: 37°C for 15 min, then 85°C for 5 sec. ChamQ SYBR Master Mix was used to perform qPCR analysis using a real-time PCR system (BIOER, Hangzhou, China) in accordance with the manufacturer's instruction. Ten *μ*M specific forward and reverse primers was used in the assay, as detailed in Table 1. Additionally, GAPDH was used as the internal reference. The qPCR procedure was performed in a thermal cycler as follows: predenaturation at 95°C for 30 sec, then denaturation at 95°C for 10 sec, and extension at 60°C for 30 sec repeated for 40 cycles. Relative gene expression was calculated using the 2^-*ΔΔ*CT^ method.

### 2.7. Flow Cytometric Analysis of CD4^+^ CD25^+^ Tregs

A 100 *μ*L aliquot of blood was incubated with Cyanine7-conjugated anti-CD45, fluorescein isothiocyanate- (FITC-) conjugated anti-CD3, Cyanine5.5-conjugated anti-CD4, and phycoerythrin- (PE-) conjugated anti-CD25 antibodies (Biolegend, San Diego, CA, United States) at 4°C for 30 min, respectively. The cell suspension was transferred into tubes and washed in phosphate-buffered saline (PBS). All stained cells were analyzed using a FACSCanto II flow cytometer (BD Biosciences) and the FlowJo software.

### 2.8. Enzyme-Linked Immunosorbent Assay (ELISA) Analysis

ELISA of rat serum samples was performed to measure the expression of interferon-gamma (IFN-*γ*), TNF-*α*, IL-1*β*, interleukin-4 (IL-4), IL-6, IL-10, IL-17A, RANKL, and OPG using the respective ELISA kits (RayBioech, Guangzhou, China), in accordance with the manufacturer's instructions. OD values at a wavelength of 450 nm were measured using a Spark 20 M multimode microplate reader (Tecan Group Ltd., Mannedorf, Switzerland). Concentrations were calculated from standard curves.

### 2.9. Western Blot Analysis

The primary antibodies used in the present study were against the following antigens: RANKL (rabbit polyclonal antibody, dilution 1 : 1000, Proteintech), OPG (rabbit polyclonal antibody, dilution 1 : 300, Abcam, Cambridge, MA, USA), and *β*-actin (internal control, rabbit polyclonal antibody, dilution 1 : 5000, Abcam). Proteins from the knee joint were separated using SDS-PAGE then transferred to polyvinylidene difluoride membranes. The intensity of each protein was normalized using *β*-actin and expressed as a proportion of the control. Analysis of Western blots was performed in accordance with a previous study [21].

### 2.10. Statistical Analysis

The experimental data are expressed as means ± standard error of the mean (SEM) and analyzed using the GraphPad Prism version 8.0 software (GraphPad, Inc., La Jolla, CA, United States). Arthritis scores, body weight, and paw edema were analyzed using repeated measures one-way analysis of variance (ANOVA). Other data were analyzed using ANOVA and *post hoc* testing or with a Student *t*-test. *P* values <0.05 were considered statistically significant.

## 3. Results

### 3.1. TSNGS Relieved RA-Like Symptoms including Paw Swelling and Clinical Score in AIA Rats

**Figure 2:**
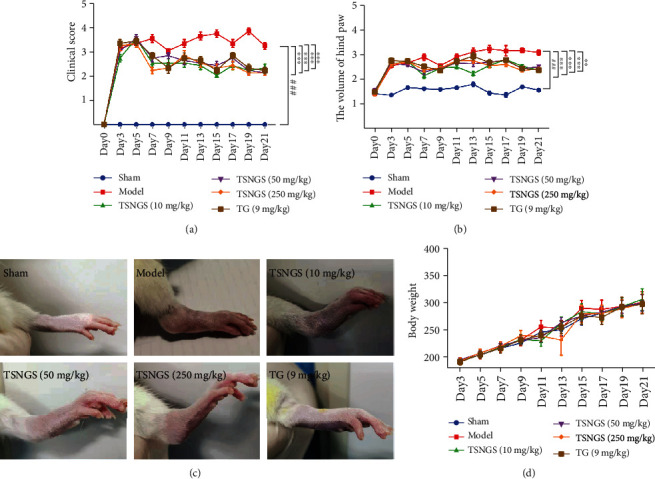
TSNGS relieved RA-like symptoms in AIA rats. (a) TSNGS significantly decreased clinical scores in AIA rats compared with model rats. (b) TSNGS markedly mitigated the swelling of the hind paws in AIA rats compared with model rats. (c) Representative images of swollen hind paws of rats in each group. (d) Rat body weights were not significantly different in each group. Results are presented as means ± SEM, *n* = 10. ^###^*P* < 0.001 vs. sham, ^∗∗^*P* < 0.01, ^∗∗∗^*P* < 0.001 vs. model.

Following subcutaneous injection of CFA in rat hind paws, compared with the control group, the volume of the hind paws increased, the inflammatory symptoms, erythema, and swelling increased significantly, relevant spontaneous activity of the rats decreased, and clinical scores were maintained at 3 to 4 points, peaking on day 3 (Figures 2(a)–2(c), *P* < 0.001), indicating the onset of arthritis.

During the progression of RA, the mean clinical score in the AIA group was significantly higher than that of the control group (Figure 2(a), *P* < 0.001). Hind paw swelling was observed to be approximately 3 mm in thickness in AIA rats (Figures 2(b) and 2(c), *P* < 0.001). Treatment with 10 mg/kg, 50 mg/kg, and 250 mg/kg TSNGS significantly reduced the clinical score and inhibited paw swelling in AIA rats during the period of the experiment (*P* < 0.01‐0.001). TSNGS at 250 mg/kg displayed protective effects comparable with those of the positive drug, TG, in the rats. No statistical difference was observed in body weight among different treatment groups (Figure 2(d)).

### 3.2. TSNGS Improved Histological Changes in Knee and Ankle Joints in AIA Rats

**Figure 3:**
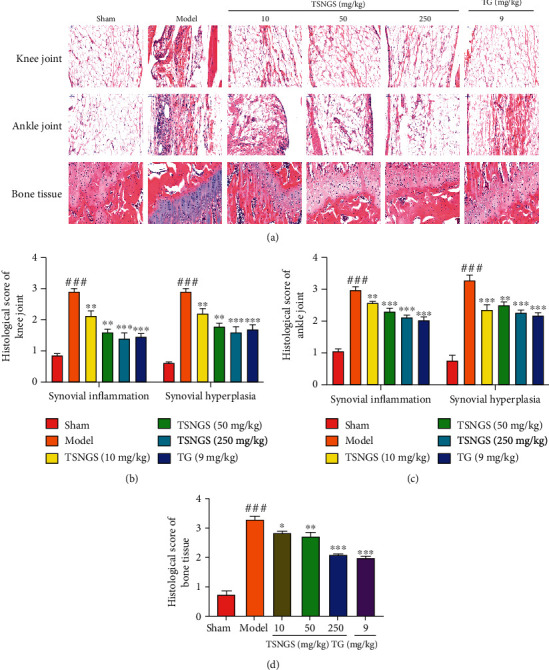
TSNGS improved histological changes in knee and ankle joints in AIA rats. (a) Representative images of H&E staining of knee joints, ankle joints, and bone tissue of knee joints in each group. Pathological severity scores of synovial inflammation and synovial hyperplasia in knee joints (b), ankle joints (c), and bone tissue (d) in rats in each group. Results represent means ± SEM, *n* = 4. ^###^*P* < 0.001 vs. sham, ^∗^*P* < 0.05, ^∗∗^*P* < 0.01, ^∗∗∗^*P* < 0.001 vs. model.

H&E staining of knee and ankle joints was conducted to evaluate inflammation and bone lesions in AIA rats. As can be observed in Figure 3, no abnormal pathological changes occurred in the knee and ankle joints of control rats. The synovial cells of the joints in the control group were smooth and continuous in morphology. Conversely, sections of AIA rats exhibited markedly severe synovitis, characterized by synovial cell proliferation and inflammatory cell infiltration into the joint cavity. The erosion of bone and cartilage and formation of pannus and small blood vessels was evident, consistent with increased synovial hyperplasia and inflammation (all *P* < 0.001). These pathological symptoms were mitigated to varying degrees after treatment with 10 mg/kg, 50 mg/kg, and 250 mg/kg TSNGS (*P* < 0.05-0.001). In particular, at a dose of 250 mg/kg, synovial cell proliferation, cartilage erosion, and infiltration of inflammatory cells were clearly attenuated and represented a better therapeutic effect than TG.

### 3.3. TSNGS Downregulated the Expression of Cytokines Mediated by Inflammation in AIA Rats

**Figure 4:**
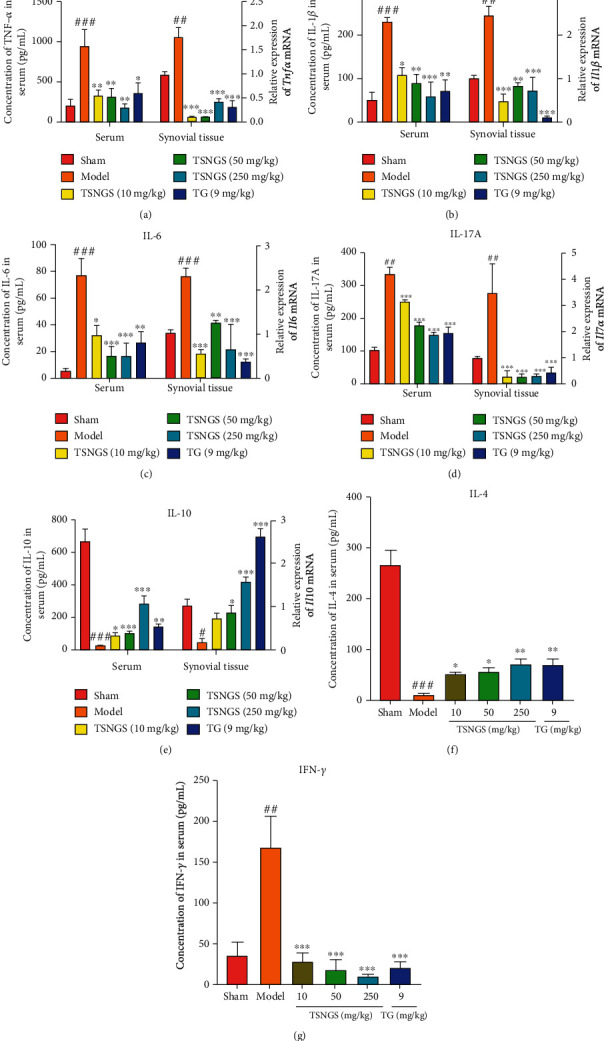
TSNGS downregulated the level of cytokines mediated by inflammation in AIA rats. The level of TNF-*α* (a), IL-1*β* (b), IL-6 (c), IL-17A (d), IL-10 (e), IL-4 (f), and IFN-*γ* (g) in rat serum or synovial tissue was measured by ELISA or qPCR. Results are presented as means ± SEM, *n* = 4. ^#^*P* < 0.05, ^##^*P* < 0.01, ^###^*P* < 0.001 vs. sham, ^∗^*P* < 0.05, ^∗∗^*P* < 0.01, ^∗∗∗^*P* < 0.001 vs. model.

ELISA and qPCR were performed to detect proinflammatory factors, including TNF-*α*, IL-1*β*, IL-6, IL-17A, IFN-*γ*, and anti-inflammatory factors IL-10 and IL-4 in rat serum and synovial tissue. The mRNA expression levels in the synovial tissue and/or serum concentration of TNF-*α*, IL-1*β*, IL-6, IL-17A, and IFN-*γ* in AIA rats were significantly higher than in the control (Figures 4(a)–4(d) and 4(g), *P* < 0.01-0.001). mRNA and protein expression levels of IL-10 and IL-4 declined in the synovial tissue and/or serum in AIA rats (Figures 4(e) and 4(f), *P* < 0.05-0.001). Treatment with TSNGS significantly inhibited inflammation by downregulation of TNF-*α*, IL-1*β*, IL-6, IFN-*γ*, and IL-17A and upregulation of IL-10 and IL-4 in the serum and synovial tissues (*P* < 0.05-0.001). A dosage of 250 mg/kg TSNGS displayed anti-inflammatory effects similar to those of TG.

### 3.4. TSNGS Increased the Proportion of CD4^+^ CD25^+^ Tregs in Peripheral Blood and the Expression of Foxp3 in Knee Joints in AIA Rats

**Figure 5:**
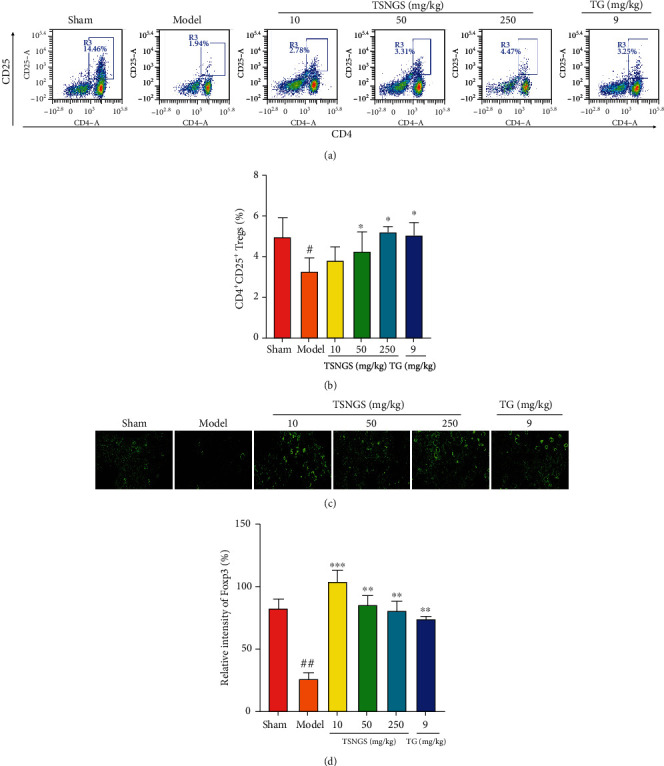
TSNGS increased the proportion of CD4^+^ CD25^+^ Tregs in peripheral blood and the expression of Foxp3 in knee joints in AIA rats. (a) Representative images of CD4^+^ CD25^+^ Tregs in peripheral blood detected by flow cytometry. (b) Quantification of the percentage of CD4^+^ CD25^+^ Tregs in peripheral blood. (c) Representative immunohistochemical staining images of Foxp3 protein. (d) Percentage of positive cells expressing Foxp3. Results represent means ± SEM, *n* = 4. ^#^*P* < 0.05, ^##^*P* < 0.01 vs. sham, ^∗^*P* < 0.05, ^∗∗^*P* < 0.01, ^∗∗∗^*P* < 0.001 vs. model.

CD4^+^ CD25^+^ Tregs are an essential subpopulation of T cells, positively expressing the transcription factor Foxp3 and yielding inflammatory factors. Functioning as an immunosuppressive group, CD4^+^ CD25^+^ Tregs maintain immunologic self-tolerance and negatively modulate the occurrence and development of immune disorders [22]. The percentage of CD4^+^ CD25^+^ Tregs declined significantly in peripheral blood (Figures 5(a) and 5(b), *P* < 0.05), accompanied by decreased levels of Foxp3 in the knee joint of AIA rats (Figures 5(c) and 5(d), *P* < 0.01). Treatment with TSNGS resulted in a significant increase in the percentage of CD4^+^ CD25^+^ Tregs and upregulated expression of Foxp3 in AIA rats (*P* < 0.05-0.001), suggesting that the promotion of a CD4^+^ CD25^+^ Treg response contributed to the immuno-inflammatory suppressive effect of TSNGS.

### 3.5. TSNGS Decreased Bone Erosion by Increasing the OPG/RANKL Ratio in AIA Rats

**Figure 6:**
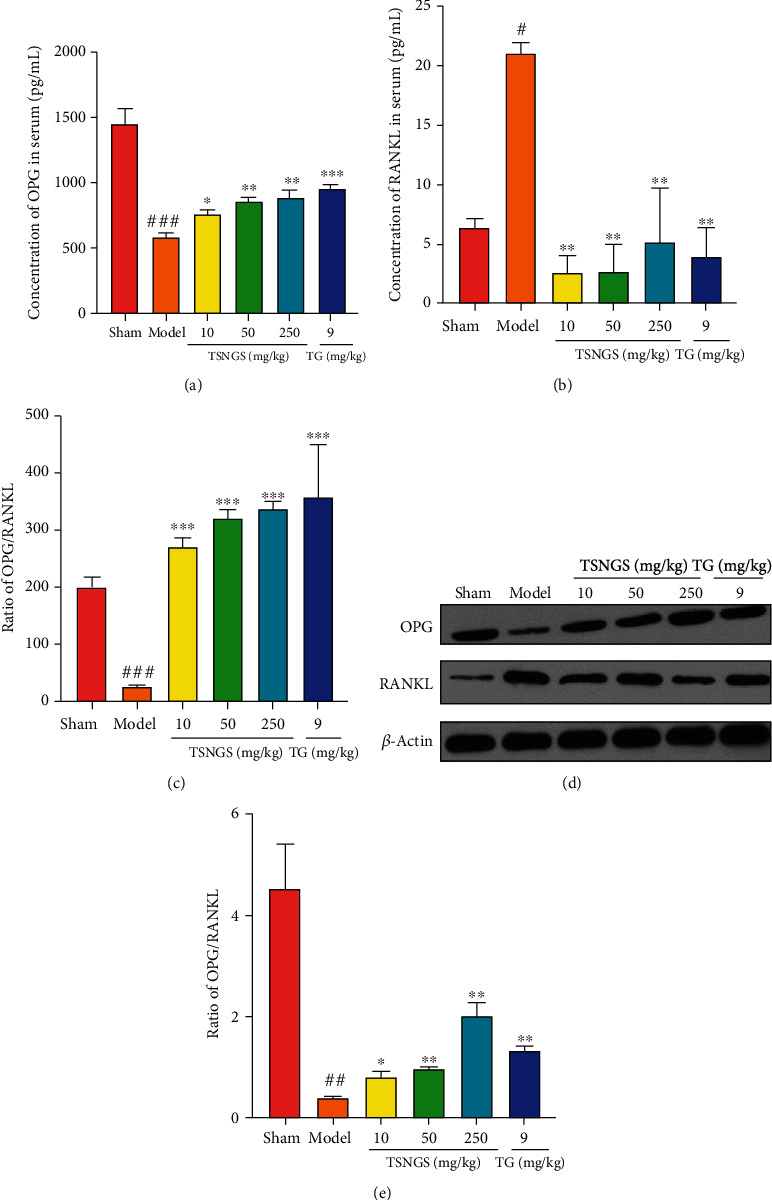
TSNGS decreased bone erosion by increasing the OPG/RANKL ratio in AIA rats. Levels of OPG (a) and RANKL (b) in rat serum were measured using ELISA. (c) Ratio of OPG/RANKL expression in rat serum. (d) Representative images of OPG and RANKL in the various groups. (e) Quantitative WB analysis. Results represent means ± SEM. *n* = 4, ^#^*P* < 0.05, ^##^*P* < 0.01, ^###^*P* < 0.001 vs. sham, ^∗^*P* < 0.05, ^∗∗^*P* < 0.01, ^∗∗∗^*P* < 0.001 vs. model.

To investigate the potential mechanism of the anti-RA effects of TSNGS on bone erosion, the OPG/RANKL ratio was measured to signify the degree of osteoclastogenesis. As shown in Figure 6, the level of OPG decreased significantly, and RANKL increased both in serum and in the knee joint, resulting in a reduced OPG/RANKL ratio compared with the control group (Figures 6(a)–6(e), *P* < 0.05‐0.001). Treatment with TSNGS resulted in a noticeable decrease in the levels of RANKL and an apparent increase in OPG levels, resulting in an increased OPG/RANKL ratio in AIA rats (*P* < 0.05‐0.001). Furthermore, the ameliorative action of TSNGS on the OPG/RANKL ratio indicated that bone erosion was equivalent to that in rats in which TG had been administered. These results indicate that TSNGS might reduce bone erosion by restoring the abnormal OPG/RANKL axis against RA injury.

## 4. Discussion

There are two major contributions the present study provides to the science of TSNGS action. To our knowledge, this is the first study to establish the beneficial effects of TSNGS against RA by alleviating inflammation, enhancing immunosuppressive function, and decreasing bone erosion. Second, following RA-associated perturbation, TSNGS was found to interfere with the counter-balancing of systemic and local inflammatory cytokines, elevation of the proportion of CD4^+^ CD25^+^ Tregs, and bone-protection due to upregulation of OPG/RANKL pathways. Therefore, these findings provide novel evidence and insights into TSNGS that clarify its therapeutic effects and possible mechanisms against RA injury.

CFA-induced RA in rats is a stable, reliable, and reproducible model of short experimental duration and similar clinical characteristics to that of human RA regarding inflammatory cell infiltration, synovitis, synovial hyperplasia, and cartilage degradation. Thus, it is widely used in the preclinical pharmacological evaluation of anti-RA drugs [23, 24]. TG, selected as a positive control drug in the present study, is routinely used for RA treatment due to its proven efficacy, relative safety, and cost-effectiveness [25]. Nevertheless, similar to DMARDs, TG has side effects, causing liver injury, gastrointestinal discomfort, and gonad toxicity [26] and therefore should be recommended only for use in patients without reproductive needs. Attention has thus focused on plant-derived products with multifactorial efficacy and relatively few side effects as potential candidate drugs for RA treatment.

Following adjuvant injection, arthritis-related symptoms were gradually exacerbated in AIA rats, in which paw swelling, clinical score, histopathological parameters, and body weight were used to assess the severity of arthritis and the beneficial effects of TSNGS. No statistical difference was observed in the body weight of AIA rats, implying that TSNGS did not produce systemic toxicity. Subsequent results demonstrated a powerful inhibition of TSNGS on paw swelling and clinical score, as well as histopathological parameters. Significantly, the effects of 250 mg/kg TSNGS on the volume of hind paws, clinical score, and histopathological parameters suggested a therapeutic potency similar or even better than that of TG, indicating that TSNGS represents a new potential therapy for the treatment of RA.

Critical proinflammatory cytokines TNF-*α*, IL-1*β*, IL-6, IL-17A, and others derived from activated macrophages and synovial fibroblasts are primary factors that cause inflammation in RA [27–30], not only in joints and synovial fluid but also in serum [31]. TNF-*α* activates the cytokine cascade in RA via stimulation of proinflammatory cytokines and inhibition of anti-inflammatory cytokines such as IL-4 and IL-10 [32]. Although five TNF inhibitors (etanercept, infliximab, adalimumab, certolizumab, and golimumab) and one IL-6 inhibitor (tocilizumab) are available for the routine clinical treatment of RA [33, 34], more than a third of RA patients do not respond to this treatment [35, 36]. Therefore, those partial responders and nonresponders to cytokine inhibitors are in great need of substitutable, effective anti-inflammatory drugs. In the present study, observation of aberrant levels of the proinflammatory cytokines TNF-*α*, IL-1*β*, IL-6, and IL-17A, besides anti-inflammatory cytokines IL-4 and IL-10 in the serum and synovial tissues of rats, indicates that the AIA model of RA was successfully established. It is evident that administration of TSNGS downregulated the expression of TNF-*α*, IL-1*β*, IL-6, and IL-17A and upregulated IL-4 and IL-10 expression, displaying a useful improvement of the inflammatory environment. Thus, these results confirm the effectiveness of TSNGS in RA therapy, illustrated by potent inhibition of the inflammatory response.

Considering that CD4^+^ T cells regulate the inflammatory environment in RA through a variety of subsets, we investigated the regulation by TSNGS of the diverse population of CD4 T cells in AIA rats. Naïve CD4^+^ T cells may differentiate into T helper cells (Th) and Tregs, characterized by cytokines IL-4 and IL-17 [37]. Tregs play a critical role in peripheral immune tolerance and inflammatory homeostasis regulators, of which the Foxp3 expression is defined as an inherent hallmark [38]. Studies have demonstrated that an imbalance in IL-17-producing Th17 and Tregs affects the pro- or anti-inflammatory T cell-mediated immune response and makes a considerable contribution to the pathological direction of RA [39]. A previous study demonstrated that IL-1*β*, IL-6, and TGF-*β* promotes the differentiation of naive T cells into Th17 cells [40] and in turn suppresses Treg differentiation, leading to homeostasis disorders. IL-17 generated by Th17 affects a variety of immune cells that activates inflammation and the differentiation of osteoclasts by induction of RANKL in the synovium [41]. Therefore, the balance between Treg and Th17 cell differentiation is defined by the patterns of cytokine production and function towards pro- or anti-inflammation. In the present study, treatment with TSNGS caused high expression of CD4^+^ CD25^+^ Tregs combined with upregulated Foxp3 expression in AIA rats, displaying excellent immune amelioration by maintaining the frequency of Tregs and the patterns of cytokine production. Therefore, an increase in the proportion of CD4^+^ CD25^+^ Tregs resulting from TSNGS treatment might be a mechanism of inflammatory inhibition in RA.

Osteoclast-mediated bone destruction that occurs through the abnormal OPG/RANKL axis plays a crucial role in RA [42, 43]. In the present study, TSNGS reduced damage to the articular cartilage and bone in AIA rats. Thus, the molecular balance between OPG and RANKL that determines the proliferation and activity of osteoclasts and osteoblasts was investigated. OPG acts as an inhibitor of RANKL, a critical factor in the formation of osteoclasts by activating osteoclasts and bone resorption. OPG reduces the interaction between RANKL and RANK and is produced by a number of cell types, including T cells, macrophages, and synovial fibroblasts in RA, thus inhibiting the formation of osteoclasts. Therefore, restoration of the OPG/RANKL ratio plays a vital role in reducing bone damage in RA. Notably, TSNGS reduced the level of RANKL and increased the level of OPG both in serum and the knee joints of AIA rats, thus raising the ratio of OPG/RANKL and so suppressing inflammation-induced osteoclastogenesis. These observations suggest that bone protection resulting from TSNGS might be associated with upregulation of OPG/RANKL.

Nevertheless, the present study has some limitations. Firstly, it is necessary to establish a greater number of RA models both *in vivo* and *in vitro* to clarify the specific molecular mechanism of the therapeutic effects of TSNGS and to analyze the key pathological genes and proteins in RA. Therefore, the extraction process or composition of TSNGS should be enhanced in the future to exert a more significant anti-RA effect in patients.

## 5. Conclusions

**Figure 7:**
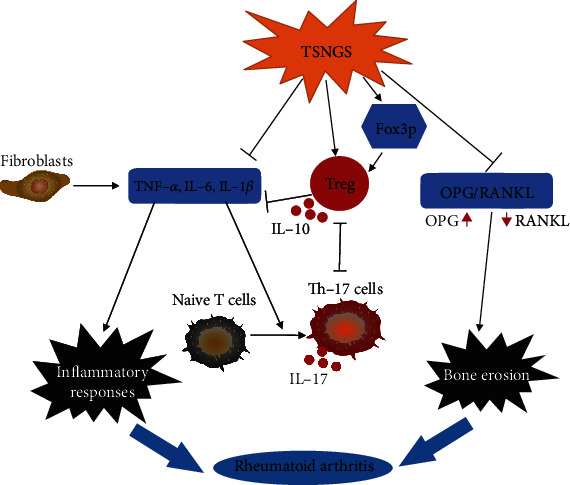
The potential mechanism by which TSNGS alleviates the pathological process of RA. TSNGS affected the inflammatory response by regulating anti-inflammatory and proinflammatory cytokines and suppressed bone erosion by activation of the OPG/RANKL pathway.

In conclusion, the present study has demonstrated that treatment with TSNGS inhibits arthritis caused by AIA, suppressing a local and systemic inflammatory response and bone erosion. The underlying mechanism of the action of TSNGS may be associated with restoration of the balance of pro- and anti-inflammatory cytokines, elevation of the frequency of CD4^+^ CD25^+^ Tregs, and reduction of the bone injury via the OPG/RANKL pathway (Figure 7).

## Figures and Tables

**Table 1:** PCR primer sequences used for qPCR.

Primer name	Primer sequence
Tnf-F	5′-CCGACTCTGACCCCCATTAC-3′
Tnf-R	5′-CCCAGAGCCACAATTCCCTT-3′
Il1*β*-F	5′-CAGGATGAGGACCCAAGCAC-3′
Il1*β*-R	5′-GTCGTCATCATCCCACGAGT-3′
Il6-F	5′-TGCCTTCTTGGGACTGATGT-3′
Il6-R	5′-TGGTCTGTTGTGGGTGGTATC-3′
Il17a-F	5′-GTCCTGAAGAGGGAGCCTGA-3′
Il17a-R	5′-GCGGACAATAGAGGAAACGC-3′
Il10-F	5′-GACAAAGGTGTCTACAAGGCCA-3′
Il10-R	5′-CAGTAGATGCCGGGTGGTTC-3′
Gapdh-F	5′-AGTGCCAGCCTCGTCTCATA-3′
Gapdh-R	5′-AGAGAAGGCAGCCCTGGTAA-3′

F: forward; R: reverse.

## Data Availability

The data used to support the findings of this study are available from the corresponding authors upon request.
